# Supplementary figures and images for: Spindle checkpoint activation by fungal orthologs of the S. cerevisiae Mps1 kinase
Source: PLoS One. 2024 Mar 26;19(3):e0301084. doi: 10.1371/journal.pone.0301084 (PMC10965065; doi:10.1371/journal.pone.0301084)

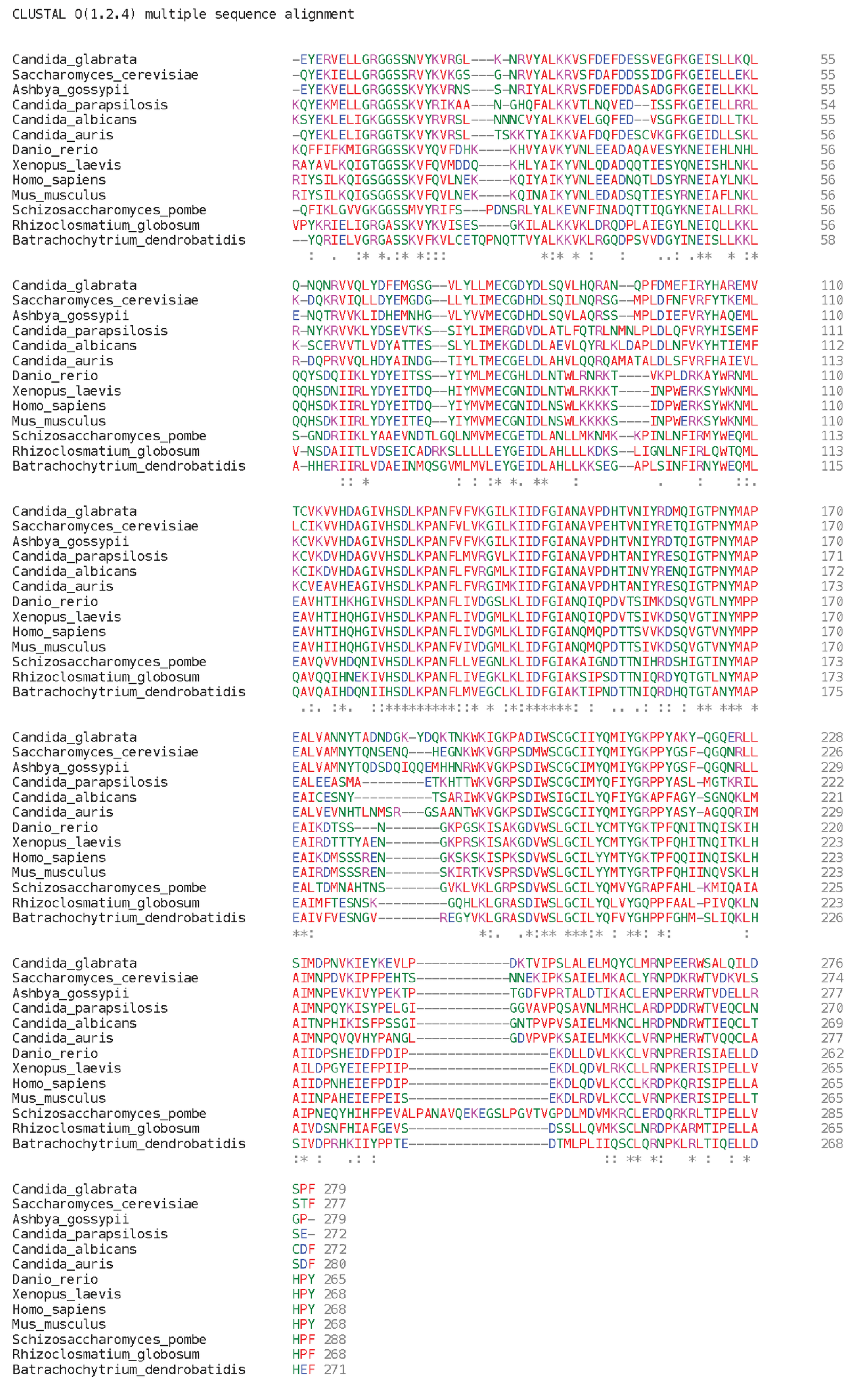

Supplement: S1 Fig — Amino acid sequence alignment of Mps1 kinase domains from indicated species. (TIF) [file pone.0301084.s001.tif]

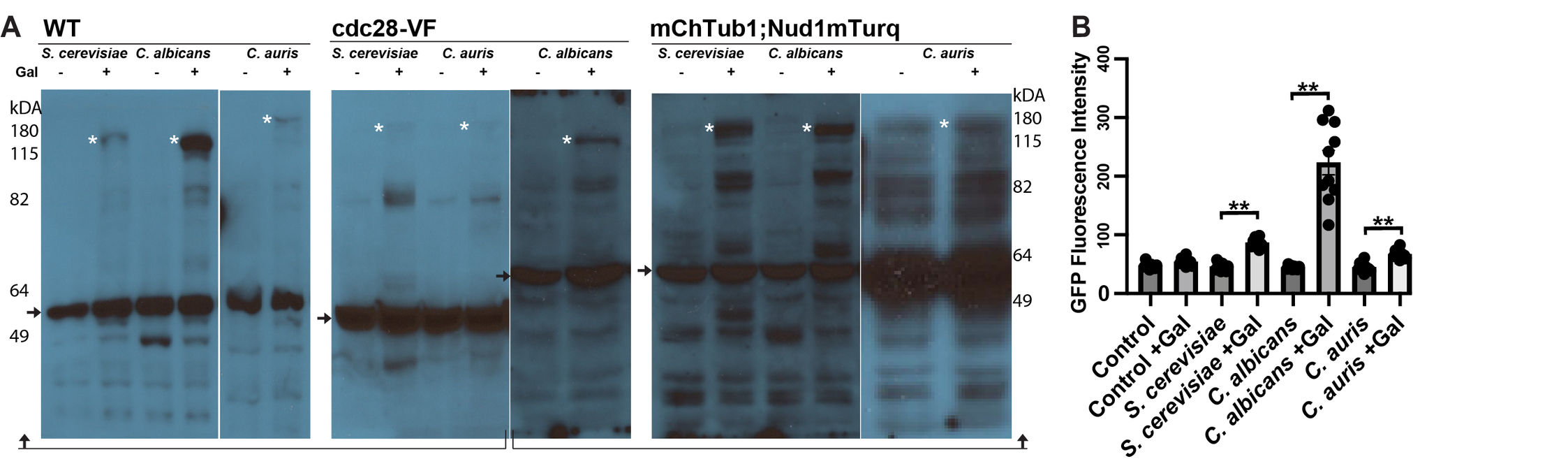

Supplement: S2 Fig — Mps1 Protein expression with (+) or without (-) galactose induction shown by A. Western blot in indicated cell background. Anti-GFP showing overexpression of indicted Gal-GFP-Mps1 construct. * = GFP-Mps1, arrows = internal control. B. GFP fluorescence intensity in nucleus measured from microscopy of cells expressing GFP-Mps1 in mCh-Tub1;Nud1-mTurq background **p<0.001. (TIF) [file pone.0301084.s002.tif]

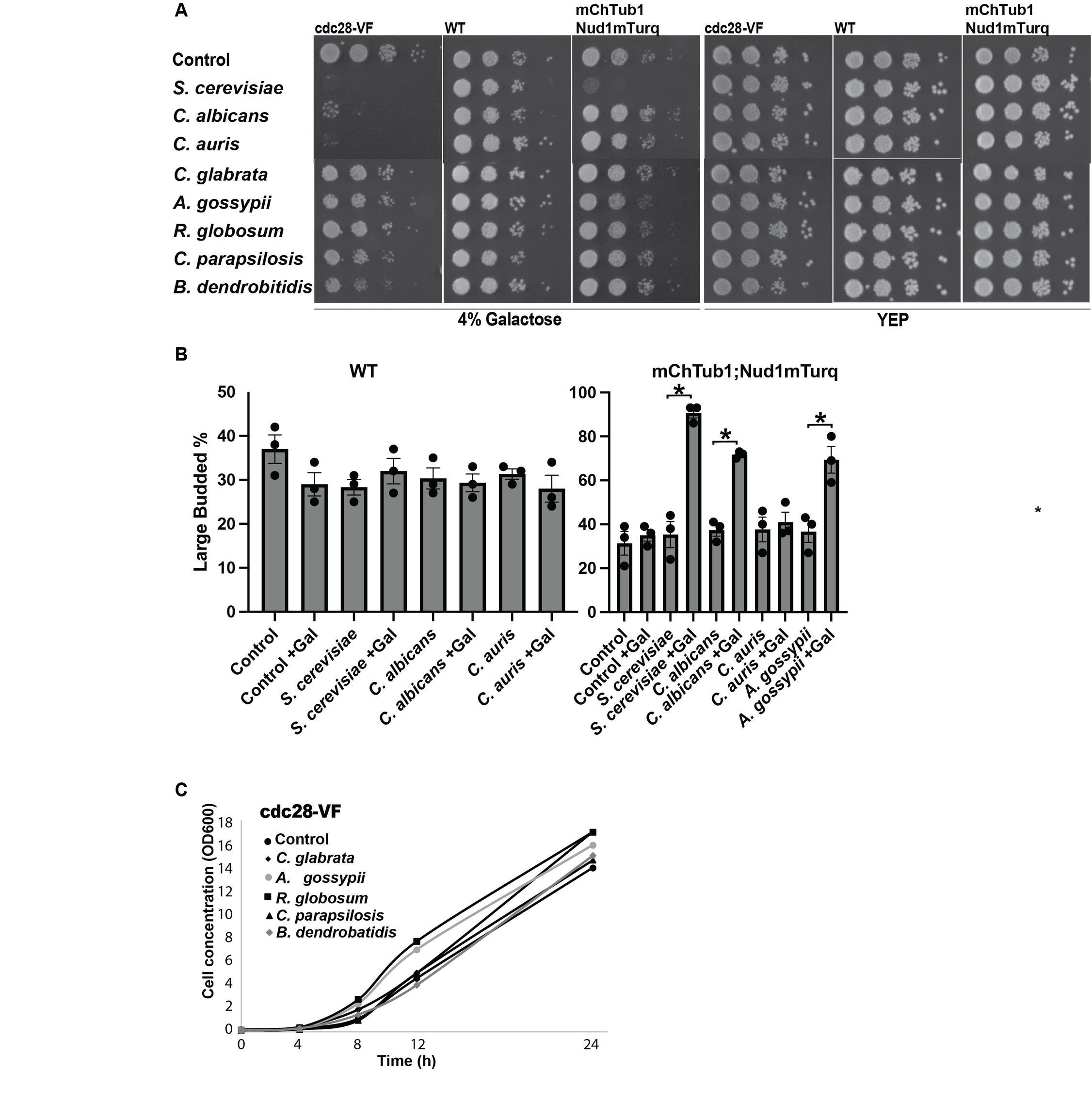

Supplement: S3 Fig — A. Growth on dilution plates of cells overexpressing indicated species of Mps1 (4% galactose) or without overexpression (YEP) in three different cell backgrounds (cdc28-VF, WT, or mChTub1;Nud1mTurq). B. Budding indices with (+Gal) or without overexpression of indicated species of Mps1 in either a wild-type or mChTub1;Nud1mTurq background. C. Growth curve (OD600) of cells overexpressing (4% galactose) indicated Mps1 in liquid culture in cdc28-VF background. *p<0.05. (TIF) [file pone.0301084.s003.tif]

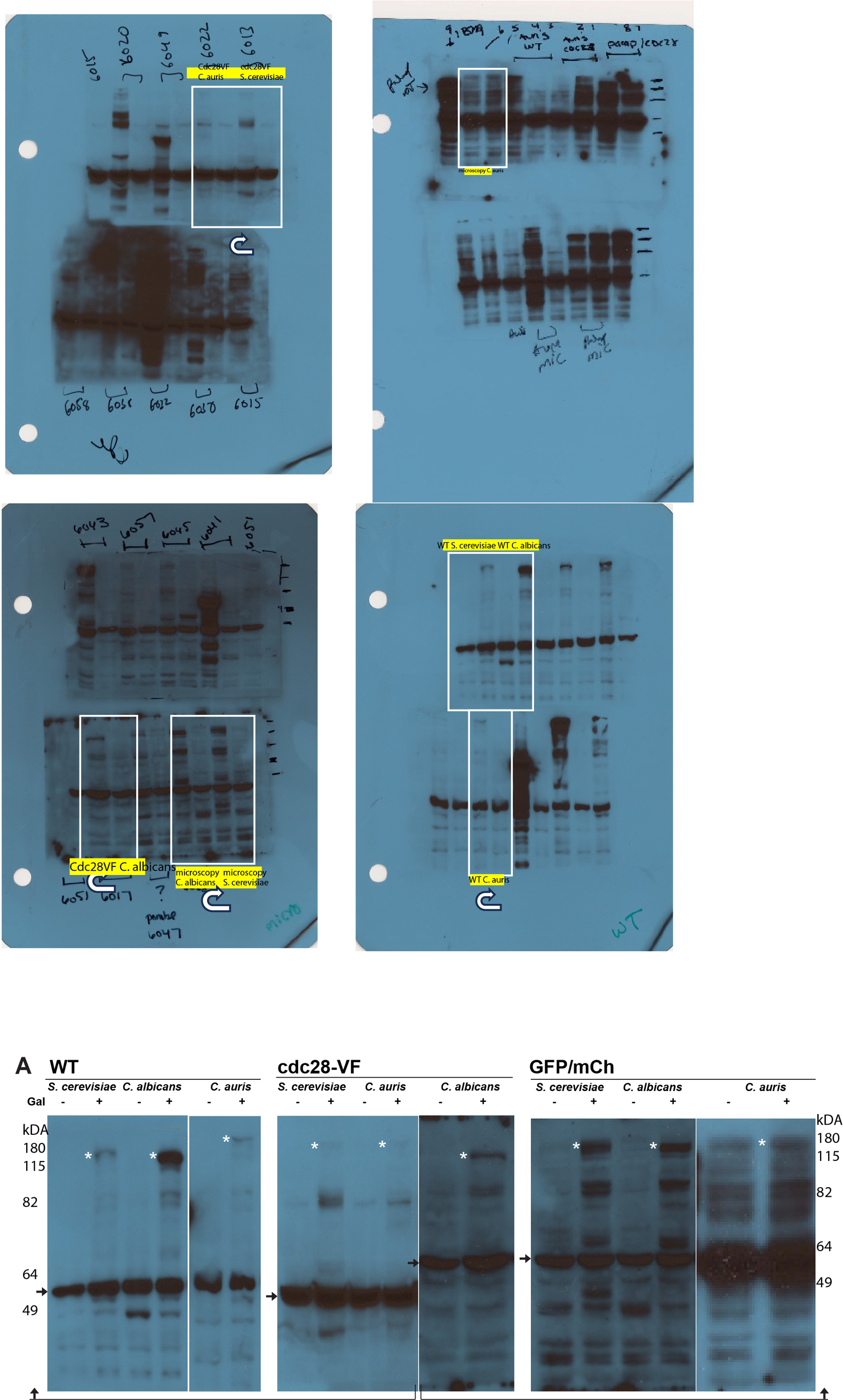

Supplement: S1 Raw images — (TIF) [file pone.0301084.s004.tif]
