# Supplementary material for: Spindle checkpoint activation by fungal orthologs of the S. cerevisiae Mps1 kinase
Source: PLoS One. 2024 Mar 26;19(3):e0301084. doi: 10.1371/journal.pone.0301084 (PMC10965065; doi:10.1371/journal.pone.0301084)
Supplement: S1 Raw data — (PDF) [file pone.0301084.s005.pdf]

Raw Data

Spindle lengths

	p<0.0001			p<0.0001		p=0.12	
	S. cerevisiae	S. cerevisiae	C	C. albicans	C. albicans	C. auris	C. auris
N	90	119		95	146	79	68
Avg	4.48	2.09		4.50051579	1.80258219	4.81920253	5.26198529
STD	1.83698	0.749706		1.6433641	1.12192418	1.72727575	1.68307082
SEM	0.195	0.069		0.169	0.093	0.196	0.206
	4.646	2.43		6.085	1.371	5.993	7.34
	4.331	1.623		3.078	1.714	1.778	5.982
	3.299	1.813		5.358	1.904	6.613	7.14
	5.85	2.155		3.237	1.814	2.602	6.765
	4.058	2.358		6.43	2.324	4.86	3.5
	4.051	2.417		5.278	2.526	7.423	4.27
	4.583	1.587		6.452	2.624	3.621	6.441
	6.03	1.718		4.989	2.025	5.283	6.493
	1.24	2.482		7.05	1.763	1.392	5.123
	1.047	1.127		8.52	0.399	5.337	4.378
	1.548	1.676		3.011	0.626	4.32	4.659
	3.985	2.54		5.361	1.57	4.728	3.216
	5.52	1.449		3.585	2.503	3.078	8.594
	4.43	2.235		4.466	2.518	4.82	2.548
	3.57	2.606		6.415	1.457	2.26	5.855
	6.461	2.852		7.069	3.218	5.395	5.255
	3.862	1.961		4.56	1.867	6.269	3.294
	4.983	2.891		4.55	0.302	5.431	9.035
	9.398	2.061		5.481	0.615	5.859	2.373
	3.722	1.331		1.076	2.068	1.356	5.339
	5.302	2.549		5.378	2.217	2.839	4.839
	4.222	2.23		5.551	2.513	6.039	6.501
	3.679	1.41		5.972	2.045	4.446	3.951
	3.694	2.201		2.934	1.331	7.563	3.405
	5.931	0.778		4.054	1.913	6.606	3.512
	5.118	2.107		2.399	2.142	5.228	5.016
	5.045	1.547		5.354	2.037	4.225	6.023
	1.06	3.027		4.55	1.225	5.573	3.67
	0.736	2.595		1.026	1.66	5.172	3.997
	5.273	1.492		4.253	1.633	4.443	6.821
	3.681	2.463		7.006	1.273	1.351	7.559
	4.088	2.628		6.338	2.439	4.892	7.321
	3.907	2.689		4.372	2.034	1.513	5.915
	1.457	1.021		4.225	1.994	5.808	6.417
	0.985	2.514		1.1	1.128	5.32	3.877
	4.454	0.986		4.009	0.72	4.855	5.478
	6.804	2.493		6.298	1.087	2.283	3.359
	1.833	2.405		4.167	0.789	5.104	4.083
	4.185	2.086		6.138	2.288	5.633	3.462
	3.413	1.963		4.187	2.139	3.389	7.225
	1.448	1.104		3.772	1.904	4.551	5.981
	1.452	3.795		3.652	1.976	8.093	2.637
	5.927	2.45		3.925	1.612	2.207	2.857
	1.057	3.721		2.742	2.096	6.577	7.372
	5.718	3.884		2.54	7.733	4.755	7.648
	5.654	3.373		4.083	1.655	6.155	2.83
	6.637	2.382		3.338	1.877	5.747	4.37
	7.087	2.653		3.388	2.467	5.777	6.622
	4.987	2.516		6.419	2.141	4.412	8.045
	4.848	3.808		6.988	1.53	4.647	6.076
	3.801	1.531		5.043	0.598	6.435	4.934
	4.555	2.996		5.456	1.901	7.216	5.123
	5.177	2.171		2.197	1.524	5.705	8.217
	1.781	3.294		4.021	1.283	3.582	8.13
	4.639	2.609		6.644	2.075	2.52	5.025
	4.952	1.99		6.295	0.821	4.661	5.065
	4.884	3.062		5.217	7.782	8.25	4.452
	4.291	3.719		5.381	1.829	6.269	5.569
	6.104	0.89		4.789	2.177	3.192	4.866
	3.639	4.75		3.798	1.654	5.519	4.958
	3.98	1.565		3.333	1.087	5.617	6.384
	1.014	3.606		1.144	1.647	6.274	5.289
	3.925	2.381		3.615	1.806	7.287	5.091
	4.414	2.263		6.988	1.231	1.449	6.165
	4.99	3.938		4.177	1.115	6.447	4.895
	5.606	3.34		6.505	1.788	5.806	5.163
	5.379	2.366		5.22	1.993	1.845	2.028
	1.142	2.046		1.588	0.858	3.741	1.992
	4.703	1.425		3.772	1.273	5.293	
	8.039	1.601		4.362	0.515	4.642	
	4.773	1.538		6.318	1.971	5.058	
	4.13	0.688		4.19	1.015	6.425	
	5.886	1.221		5.869	2.051	6.479	
	5.7	1.411		5.765	2.986	3.378	
	5.708	1.377		5.422	2.254	2.896	
	5.469	1.521		3.892	9.428	7.381	
	3.846	2.307		1.143	2.811	6.771	
	7.358	0.572		5.048	1.943	4.714	
	4.502	1.312		2.538	1.412	2.244	
	3.608	1.972		3.949	3.614		
	7.587	2.218		4.213	3.185		
	8.13	1.609		4.164	2.791		
	5.149	2.27		6.301	2.222		
	5.684	1.9		1.596	1.866		
	1.045	1.881		4.757	2.014		
	6.214	1.563		4.307	0.917		
	7.184	1.386		6.573	1.706		
	6.051	2.233		1.689	1.585		
	6.449	2.143		6.897	1.873		
	5.621	1.293		4.735	1.498		
		0.92		1.347	1.255		
		2.275		3.571	1.628		
		1.606		5.251	1.9		
		1.607		2.633	1.292		
		1.204		3.627	0.719		
		1.927			0.994		
		1.401			1.806		
		1.432			1.025		
		1.475			1.292		
		2.095			2.009		
		1.633			1.463		
		1.884			0.7		
		1.516			1.409		
		1.694			0.894		
		2.242			2.076		
		2.202			1.372		
		1.719			1.238		
		2.382			1.065		
		2.148			1.881		
		1.276			0.977		
		1.432			1.262		

2.082	1.374
1.759	2.152
2.63	1.845
2.152	1.023
2.01	1.047
1.827	1.156
2.016	1.289
2.163	1.202
	1.639
	2.121
	1.456
	1.362
	1.175
	2.267
	1.98
	2.822
	1.695
	1.375
	0.912
	1.29
	2.444
	1.859
	1.161
	1.735
	1.495
	2.542
	1.633
	1.64
	1.408
	0.878
	2.618
	0.867
	1.677
	1.361
	1.515

Liquid Growth

growth curve.MPS1

	0	4	8	12	24
wt	0.152	0.25	0.635	4.1	11.93
cer	0.179	0.327	0.602	1.55	2.84
pom	0.167	0.187	0.649	4.2	13.6
alb	0.172	0.315	0.607	2	11
mg	0.155	0.273	1.089	7	16
parap	0.13	0.223	0.97	5	14.6

6 2 21-growth curve.MPS1

CFU	0	4	8	14	24
wt	20	22	650	3200	5000
cer	30	3	12	15	40
Kin.Mut	45	35	290	1600	3500
Dend	23	46	300	1800	3700
Auris	16	2	10	10	20
Human	35	90	620	3000	4500
Glabrata	38	40	490	1800	4200
Ashbya	42	64	360	2800	4000
R.Globosum	36	65	520	2800	4200

Growth curve OD600 combined

	0	4	8	12	24
Ctrl	0.116	0.278	1.1075	4.55	13.965
S. cerevisiae	0.1645	0.325	0.651	1.125	2.13
C. albicans	0.172	0.315	0.607	2	11
C. auris	0.105	0.208	0.4	0.65	0.9

6 27 21-growth curve.MPS1 copy

OD600	0	4	9	12	24
wt	0.08	0.306	1.58	5	16
cer	0.15	0.323	0.7	0.7	1.42
Kin.Mut	0.092	0.299	1.31	3.46	14
Dend	0.13	0.32	1.48	4	15
Auris	0.21	0.416	0.8	1.3	1.8
Human	0.131	0.357	1.45	4	16
Glabrata	0.192	0.421	1.9	5	17
Ashbya	0.103	0.3	2.4	7	15.9
R.Globosum	0.17	0.373	2.75	7.7	17

growth curve.mps1-2 2

CFU-YPD

	0	4	8	12	16
wt	6	38	108	560	780
cer	9	7	4	5	7
pom	7	20	82	600	760
alb	8	4	18	95	120
mg	8	26	116	450	750
parap	7	16	80	400	690

	0	4	8	12	24
Ctrl	0.116	0.278	1.1075	4.55	13.965
C. glabrata	0.192	0.421	1.9	5	17
A. gossypii	0.103	0.3	2.4	7	15.9
R.globosum	0.17	0.373	2.75	7.7	17
C. parapsilos	0.13	0.223	0.97	5	14.6
B. dendrobat	0.13	0.32	1.48	4	15
M. globosa	0.155	0.273	1.089	7	16
M. globosa K	0.092	0.299	1.31	3.46	14

Bud Index

**Cdc28-vf**		percent lg budded						Number of cells counted		
		ASF 25 Marc	ASF 22 April	ASF 27 April	Avg			ASF 25 Marc	ASF 22 April	ASF 27 April
y4451	Ctrl	37	54	34	41.6666667			60	76	59
"	Ctrl + Gal	31	47	21	33			55	59	72
y1844	Cdc28vf	28	49	41	39.3333333			80	81	51
"	Cdc28vf + Ga	33	54	42	43			66	56	73
y6013	S. cerevisiae	33	42	38	37.6666667			54	65	82
"	S. cerevisiae	52	86	82	73.3333333	p<0.05		56	58	54
y6017	C. albicans	47	39	37	41			79	66	68
"	C. albicans +	54	93	86	77.6666667	p<0.05		78	56	63
y6015	C. auris	46	46	52	48			83	61	63
"	C. auris + Ga	63	81	77	73.6666667	p<0.05		73	57	52
y6051	S. pombe	50	49	56	51.6666667			76	51	68
"	S. pombe + G	55	89	90	78		0.088	72	55	51

**w303**		ASF 8 July 20	ASF 13 July 2	ASF 20 July 2022			ASF 8 July 20	ASF 13 July 2	ASF 20 July 2022
y4451	ctrl	38	42	31	37		66	62	57
"	ctrl+Gal	25	28	34	29		73	75	65
y6006	S. cerevisiae	29	25	31	28.3333333		73	65	59
"	S. cerevisiae	37	27	32	32		62	78	71
y6008	C. albicans	29	27	35	30.3333333		65	70	55
"	C. albicans +	29	26	33	29.3333333		52	62	60
y6066	C. auris	33	29	32	31.3333333		61	61	77
"	C. auris + Ga	34	26	24	28		56	53	59
y6011	C. auris (B)	19	27	30	25.3333333		53	67	78
"	C. auris (B) +	31	32	23	28.6666667		64	72	64

**mChTub1;Nud1mTurq**		30-Aug-22	31-Aug-22	1-Sep-22			30-Aug-22	31-Aug-22	1-Sep-22
y5980	ctrl	39	34	21	31.3333333		57	107	76
"	ctrl+Gal	39	30	36	35		56	66	70
y6026	S. cerevisiae	38	44	24	35.3333333		73	57	71
"	S. cerevisiae	93	93	86	90.6666667	p<0.01	60	59	52
y6028	C. albicans	41	39	32	37.3333333		73	72	85
"	C. albicans +	73	72	70	71.6666667	p<0.01	60	65	60
y6067	C. auris	40	46	27	37.6666667		62	71	71
"	C. auris + Ga	36	37	50	41		59	60	62
y6045	A. gossypii	40	43	27	36.6666667		58	60	63
"	A. gossypii +	69	59	80	69.3333333	p<0.05	61	68	66
y6041	M. globosa	36	36	29	33.6666667		59	61	79
"	M. globosa +	36	35	33	34.6666667	p=0.7	63	63	75
y6080	M. globosaKI	42	50	47	46.3333333		60	58	77
"	M. globosaKI	34	34	40	36	p<0.05	59	62	70

Fluorescent Intensity

large budded, short spindle

10x10 pix 0.65x0.65 um

	5980 ctrl	5980 ctrl GA	6026 Scer	6026 Scer G/	6028 Calb	6028 Calb G/	6067 Cauris	6067 Cauris (	6041 Mglob	6041 Mglob (	6080 Mglobk	6080 MglobKD GAL
1	50.218	53.93	52.711	81.6	46.66	184.89	50.98	82.81	52.56	290.01	46.656	190.544
2	45.236	55.45	37.833	73.87	50.13	296.16	60.89	68	54.78	300.93	56.83	225.11
3	58.555	54.07	40.606	96.59	45.73	258.68	42.28	64.7	46.49	300.33	45.89	186.11
4	40.7	51.49	37.667	80.28	40.92	241.844	41.69	62.76	49.32	164.29	44.13	167.278
5	45.65	45.12	43.989	94.72	41.03	292.51	52.07	71.6	42.19	284.68	56.57	90.133

6	43.25	51.59	46.25	85.1	47.284	160.17	33.16	66.76	42.37	231.3	47.76	204.389
7	49.25	66.72	39.722	79.27	45.46	116.79	41.3	73.59	46.97	148.25	39.19	164.06
8	48.13	46.76	57.41	98.42	41.97	311.989	39.13	63.37	37.39	231.91	48.78	150.256
9	46.72	54.3	50.136	84.09	41.36	192.09	41.6	60.3	44.24	410.53	44.61	226.089
10	48.16	60.2	51.08	89.87	45.23	176.82	45.39	56.6	42.83	233.24	47.46	130.42
average	47.5869	53.963	45.7404	86.381	44.5774	223.1943	44.849	67.049	45.914	259.547	47.7876	173.4389
		p=0.0912		p<0.0001		p<0.0001		p<0.0001		p<0.0001		p<0.0001
