# Supplementary material for: Spindle checkpoint activation by fungal orthologs of the S. cerevisiae Mps1 kinase
Source: PLoS One. 2024 Mar 26;19(3):e0301084. doi: 10.1371/journal.pone.0301084 (PMC10965065; doi:10.1371/journal.pone.0301084)
Supplement: Attachment — Submitted filename: PLOS-Winey_review.docx [file pone.0301084.s006.docx]

The manuscript by Fabritius et al. describes the phenotype of overproduction of Mps1 fungal orthologs in budding yeast. The authors have found that heterologous expression of the *MPS1* orthologs from *C. albicans* and *C. auris* with the *GAL* promoter leads to budding yeast cell lethality, the phenotype of which is comparable to that of overproduction of the endogenous Mps1. By observing the mitotic spindle morphology, the authors demonstrate that the cell growth defect caused by Mps1 and Mps1 ortholog overproduction is likely due to the activation of the mitotic spindle checkpoint. Surprisingly, overproduction of Mps1 orthologs from other related yeast species tested in this work show no detectable cell growth defect. Thus, the main conclusion of this work is limited, i.e. Mps1 from *C. albicans* and *C. auris*, which are closely related to budding yeast, function wise is comparable to budding yeast Mps1, whereas the other Mps1 orthologs tested by the authors are not. The experiments were conducted in a seemingly rigorous fashion, however, there is no experimental evidence presented in this work to support their main claim that heterologous expression of Mps1 orthologs in budding yeast can serve as a platform for drug screening.

The figure legends are very sketchy, they should be self-evident and detailed enough for the reader to understand the experiments.

Fig 2A should include a YEP panel as shown in Fig S3A.

Fig 3 needs quantification of spindle length and the number of cells observed.

Fig S2. Panel B is not the quantification of panel A, please explain the logic.
