# Supplementary material for: Spindle checkpoint activation by fungal orthologs of the S. cerevisiae Mps1 kinase
Source: PLoS One. 2024 Mar 26;19(3):e0301084. doi: 10.1371/journal.pone.0301084 (PMC10965065; doi:10.1371/journal.pone.0301084)
Supplement: Attachment — Submitted filename: Response to Reviewers.docx [file pone.0301084.s007.docx]

**Mark Winey**
Dean

February 26, 2024

Michael Polymenis, Ph.D.

Academic Editor

PLOS ONE

Dear Michael,

Thank you for your attention to our manuscript and for sharing the supportive and helpful feedback from the reviewers. Below we have listed the reviewer’s comments in italics followed by our answer to the comment or how we have addressed the issues preceded by “AU:.”

We have also submitted the requested copy of the revised manuscript with track changes indicated, and an unmarked version of the revised manuscript.

*Reviewer #1: The manuscript by Fabritius et al. ….. there is no experimental evidence presented in this work to support their main claim that heterologous expression of Mps1 orthologs in budding yeast can serve as a platform for drug screening.*

AU: We agree that there is no evidence directly supporting the claim of a drug screening platform. We present this as an idea of how to extend or apply these results.

*The figure legends are very sketchy, they should be self-evident and detailed enough for the reader to understand the experiments.*

AU: The figure legends have been expanded to be more detailed.

*Fig 2A should include a YEP panel as shown in Fig S3A.*

AU: A YEP panel has been added to Fig 2A.

*Fig 3 needs quantification of spindle length and the number of cells observed.*

AU: Quantification of spindle length and number of cells observed has been added to Fig. 3 and to the methods section, respectively.

*Fig S2. Panel B is not the quantification of panel A, please explain the logic.*

AU: Fig. S2 Panel B is not the quantification of A, panel B reports GFP signal. We report two different ways to document MPS1 expression, and this has been clarified in the revised figure legend.

Reviewer #2/Kevin Hardwick:

*Figure 1: Is part B necessary here? I am not convinced it is.*

AU: Figure 1, part B has been removed, as it was determined to be unnecessary

*Figure 2*

*A: just a few colonies are growing in the strain expressing C. albicans. Is this reproducible? I wonder if these rare isolates are really expressing Mps1 still? They may account for most of the liquid growth in panel B (after 12 hours).*

AU: The few colonies growing in the plate assays of C. albicans is reproducible, and we can’t be certain that they’re still overexpressing MPS1. While possible that these “escapers” may be the cause of growth in the liquid growth assay (Fig2B) after 12 hours, it seems unlikely because of the short timeframe of 12 hours in liquid culture versus days on the plates. Nonetheless, this has been noted in the text.

*C. How does this VF dataset relate to S3 Fig.B, where apparently no large-budded arrests are seen in wild-type cells? The latter seems odd – wouldn’t mitotic delays be expected here too?*

AU: While the VF strains (where CDC28 is insensitive to Swe1 inhibition) overexpressing MPS1 present a large-budded arrest (as well as reduced grown on plates and in liquid), this is not visible in a cycling population in a wild-type background expressing MPS1. In wild-type cells, fully functional Cdc28 allows the cells to bypass the arrest caused by increased MPS1 after a few hours. Our assays are performed with asynchronous cells growing 5 hours or more, allowing plenty of time for the cells to bypass the arrest with the aid of Cdc28. When Cdc28 is not regulated properly (i.e. cdc28-VF), the arrest persists, and we see the growth phenotypes, including large-budded arrests and decreased growth on plates and in liquid media.

*D: could the position of 1C and 2C control peaks (for the VF strain) simply be labelled here, on each plot? ‘DNA content index’ isn’t so helpful.*

AU: The peaks in the cytometry plots have been labelled 1C and 2C for clarification.

*Figure 3. Scale bars should be added for these IF images.*

AU: Scale bars have been added to microscopy images.

*Fig. S2*

*A: These blots are not ideal and in too many pieces. These should be improved or deleted.*

*The position of relevant molecular weight markers should be labelled.*

AU: While the presentation of the Western blots is not ideal, we believe the information may be valuable to some readers and would like to share it as part of the supplemental information. Original full blots have been provided to the journal; the position of relevant molecular weight markers have been added.

*B: The GFP quantitation, in panel B, more closely matches the western results from wild-type cells. Was expression of the 5 other Mps1-GFP fusion proteins detected (by western), in the VF strains that did not show significant growth defects (Cg, Ag, Rg, Cp, Bd)?*

AU: GFP expression was sometimes variable among the strains, but we are hoping to show that the GFP-MPS1 expression is increased in the +Gal conditions. We have noted in the text that expression may be variable. Expression of the other 5 Mps1-GFP fusion was detected in some (but not all) VF strains that did not show significant growth defects. This was sometimes variable, and since there was not a clear growth phenotype, we did not further pursue establishing expression.

*Fig. S3*

*A: why does the wild-type strain over-expressing ScMps1 grow so well, when the Nud1/Tub1 equivalent does not?*

AU: The better growth of the wild-type strain overexpressing ScMPS1 than the Nud1/Tub1 strain overexpressing ScMPS1 may be due to a synthetic phenotype caused by the addition of fluorescent markers to cytoskeletal components.

*B: why no large budded delay/arrest in GAL, in these WT strains? Cf Fig.2C and Nud1/Tub1 panel.*

AU: Please see the above explanation concerning Fig 2C.

*C: the gossypii-expressing strain delays/arrests as large budded (in the tagged Nud1/Tub1 strain) and colonies grow slowly. This doesn’t seem to happen in wild-type or VF. Could it be a synthetic phenotype with the tagged cytoskeletal markers (not fully functional)? Why is there no large-budded delay in the Nud1/Tub1 strain expressing C. auris?*

AU: The gossypii-expressing Mps1 Nud1/Tub1 tagged strain being delayed/arresting as large- budded cells (but not in other backgrounds) may be due to a synthetic phenotype with the tagged cytoskeletal markers. It is unclear why *C. auris* Mps1 overexpression doesn’t cause a large-budded arrest in the Nud1/Tub1 background (while others do); but this lack of arrest is consistent with wild-type growth in the plate assay. It’s also surprising that there is an arrest in the other strains, as the plate growth phenotype would not suggest this result. However, this may be a short term arrest that is overcome in the longer-term plate growth assay – this arrest may be due to a synthetic phenotype with the Nud1/Tub1 tagging.

*B.d. overexpression looks unhappy, but only in the VF. Any large budded arrest there?*

AU: The B.d. Mps1 overexpression in the cdc28-VF background was not checked for a large-budded arrest.

Please amend the Funding Statement as follows:

This work was supported by UC Davis and P01 NIH (GM105537 to M.W.). The funders had no role in the study design, data collection and analysis, decision to publish, or preparation of the manuscript.

We appreciate the opportunity to submit a revised manuscript. We hope that we have been able to sufficiently address to the reviewer’s comments such that the manuscript is now acceptable for publication.

Please let us know if you have any further questions.

Sincerely,

Amy Fabritius, Ph.D., Project Scientist	Mark Winey, Ph.D., Distinguished Professor

Molecular and Cellular Biology, UC Davis	Molecular and Cellular Biology, UC Davis
